# Combination of Autofluorescence imaging and salivary protoporphyrin in Oral precancerous and cancerous lesions: Non-invasive tools

**DOI:** 10.4317/jced.52100

**Published:** 2015-04-01

**Authors:** Jasdeep Kaur, Reinhilde Jacobs

**Affiliations:** 1 BDS, MSc, OMFS IMPATH research group, Dept. Imaging & Pathology, Faculty of Medicine, University of Leuven and Oral & Maxillofacial Surgery, University Hospitals Leuven, Leuven, Belgium; 2 DDS, MSc, PhD, Dr h.c, OMFS IMPATH research group, Dept. Imaging & Pathology, Faculty of Medicine, University of Leuven and Oral & Maxillofacial Surgery, University Hospitals Leuven, Leuven, Belgium

## Abstract

**Background:**

Normal and cancerous tissues have distinct auto-fluorescence properties because of differences in their biophysical and biochemical agents. Scientific evidences related to diagnostic fluorescence imaging for detection of oral precancerous and cancerous lesions are very limited.

**Objectives:**

The aim of this study was to find out potential relationships between serum, salivary and tissue protoporphyrin IX ( PX) levels in subjects with or without oral precancerous and cancerous lesions. Also , to find out diagnostic value of fluorescence imaging (VELscope® system , LED Dental Inc., White Rock, B.C.) and salivary protoporphyrin IX (PX) in oral precancerous and cancerous lesions. Furthermore this study attempts to find out diagnostic value of the combination of approaches of fluorescence imaging and salivary protoporphyrin for detection of oral precancerous and cancerous lesions.

**Material and Methods:**

The study sample comprised 3 test groups, with biopsy confirmed precancerous (leukoplakia and lichen planus) and cancerous lesions (squamous cell carcinoma) and one control group of 25 healthy individuals. To find out sensitivity and specificity, another 100 patients presenting for routine dental care were selected and clinical examinations were followed by fluorescence imaging and normal photography, which were finally confirmed by biopsy. The clinical and histopathogical examinations were done in conjunction with photography of the oral cavity using digital camera and fluorescence imaging. Serum, tissue and salivary protoporphyrin (PX) levels were measured.

**Results:**

Using fluorescence imaging, oral cancerous and precancerous lesions showed deep purple to deep brown and dark green colour respectively, while normal tissues showed pale green colour in contrast. The PX levels in serum, salivary and tissues were significantly higher in precancerous and cancerous lesions as compared to normal healthy tissues. Salivary and serum PX levels were highly correlated in all groups. The sensitivity and specificity to the discrimination of precancerous and cancerous lesions from the healthy tissues were higher by combination approaches of salivary protoporphyrin X and VELscope® system as compared individual approach.

**Conclusions:**

Combination approach of salivary protoporphyrin X and VELscope® system are more sensitive and specific to discriminate precancerous and cancerous lesions from the healthy tissues as compared to individual approach. Further studies are required on large samples of oral precancerous and cancerous lesions to test sensitivity and specificity and thus validate the clinical applicability of fluorescence imaging in (pre)cancerous diagnostics.

** Key words:**Fluorescence imaging, oral cancerous, precancerous, protoporphyrin IX, saliva.

## Introduction

Head and neck cancers constitute a large proportion of cancers in India accounting for 23% of all cancers in males and 6% in females (1). The vast majority of these cancers are related to consumption of tobacco, alcohol, poor hygiene, diet and viral infections (2,3). Early detection and diagnosis of premalignant and malignant oral mucosal lesions have the potential to significantly reduce patient morbidity and mortality. Unfortunately, the clinical appearance of these lesions can often be so understated that these may remain unnoticed or ignored by patients or dentists. Furthermore, the lesions can be difficult to clinically differentiate from more common benign tissue changes, such as those associated with infectious or inflammatory sources or post-surgical alterations (4-6). A non-invasive and reliable adjunctive tool that directs clinicians towards sites suspicious for pre-malignancy could lead to significant advancements in early detection and diagnosis of oral precancerous and cancerous lesions.

In recent years, visual tools for helping in the diagnosis of oral cancer have made important advancements by adding luminous detection systems (chemoluminescence and tissue fluorescence techniques) for improving detection and increasing the capacity to identify potentially malignant lesions (6-12). The VELscope® system (Visually Enhanced Lesion Scope; LED Dental Inc., White Rock, B.C.) is a simple handheld device detecting the loss of fluorescence in visible and non-visible high-risk oral lesions by applying direct fluorescence. The loss of fluorescence reflects a complex mixture of alterations to the intrinsic tissue distribution of fluorophores (7-13). Hence, early biochemical changes are detected by their more evident appearance, permitting early detection of pathological lesions (14). In the past, few studies have evaluated this system (11-12). Reported sensitivity values ranged from 97% to 98% and specificity from 94% to 100% (8-19). Preliminary results were promising, yet information regarding the ability of the VELscope to identify premalignant regions within Class II (innocuous) lesions or to reveal lesions otherwise visually undetectable is limited.

The autofluorescence lifetimes of normal, precancerous, and cancerous tissues are different because of varying biochemical changes, enabling characterization of the various lesions. Protoporphyrin IX (PX), an important chemical of heme synthesis, is one kind of natural fluorophores in human cells (13). One of the rate limiting steps in the heme biosynthesis pathway is the conversion of protoporphyrin IX (i.e. the photosensitizer) to heme, which is controlled by a rate-limiting enzyme, ferrochelatase (FC), through adding a ferrous iron to PX (14). Because of a lack of ferrochelatase, cancerous cells may accumulate more PX, presenting significantly longer fluorescence lifetime than that of normal cells. So, till date no study was published on ferrochelatase and protoporphyrin IX levels in oral precancerous and cancerous lesions and their relation to VELscope imaging. Hence, the aim of this study was to find out the relationship between fluorescence imaging and protoporphyrin IX levels in tissues, serum and saliva of oral precancerous and cancerous patients. This study tried to validate a simple non-invasive approach used for the diagnosis of occult oral diseases.

## Material and Methods

-Subjects: Twenty five squamous cell carcinoma (SCC), 30 leukloplakia (OLU, age 56-75 years; M:F; 15:15) and 25 lichen planus patients ( aged; 54-76 years; M:F;13:12) with biopsy-confirmed and 25 normal healthy ( age 54-75 years, M:F; 13:12) were recruited, after taking informed consent based on the definition of oral cancer and precancerous lesions by the World Health Organization (9). Ethical approval was taken from centre under JBR society (Ethical approval number JBR#1238). An additional 100 patients (aged; 45-75 years, M:F; 52:48) for testing sensitivity and specificity presenting for routine dental care were selected. Patients with history of chemotherapy, radiotherapy, oncological surgery, obesity, systemic diseases, bronchial asthma, drug allergies, alcohol abuse and smoking were excluded from the study.

-Clinical and Laboratory investigations: All subjects underwent comprehensive clinical examinations, followed by fluorescence imaging and white light photography. Positive imaging areas were referred for confirmation by scalpel biopsy and further histopathological examinations. Photographs of oral cavity tissues were taken with a digital camera and VELscope (LED Med. Inc., White Rock, BC0 (8-12).

-VELscope examination: Room lights were dimmed and the oral cavity was re-examined using the VELscope. Visual fluorescence retention (VFR) and visual fluorescence loss (VFL) were assessed and mapped on a data collection sheet. VFL was defined as mucosal sites which showed a reduction in the normal pale green auto fluorescence (appeared dark) when compared to adjacent tissues and as an anatomic control compared to tissues on the contra lateral side. The examiner’s clinical impression/clinical diagnosis of areas exhibiting VFL were recorded. VFL was considered a positive VELscope finding and, therefore, necessitated biopsy. The VELscope images and white digital images were compared.

-Oral biopsy and tissue preparation: Oral biopsies of lesions as well as normal healthy tissues were taken for analysis of PX by one of the oral surgeon. Two-mm punch biopsies were trimmed to remove a large part, frozen in liquid nitrogen, and homogenized at 2,000 r.p.m. with the Microdismembrator U (Merck KgaA, Darmstadt, Germany ) for 2 min. Chloroform: methanol (2:1 vol/vol, Merck KgaA, Darmstadt, Germany) mixture was used as an extraction medium. Mixtures were centrifuged for 15 min at 15,000 r.p.m., and the supernatant was collected for further analysis. A second extraction cycle was performed by adding the same amount of chloroform: methanol (2:1 vol/vol) mixture to the pellet. This mixture was vortexed for at least 5 min until a new suspension was formed and centrifuged for 15 min at 15,000 r.p.m. Subsequently, the supernatant was collected and added to the supernatant from the first extraction cycle. The PpIX extraction procedure was identical to the procedure used by previous study (15). All these steps were performed in a dark laboratory environment taking ultimate care to prevent exposure of the specimens to light.

-Serum and salivary protoporphyrin analysis: Approximately 5 ml of blood sample was drawn under aseptic precautions and centrifuged for 5 min to obtain serum which was stored at –65° C in sterile vials. During the examination paraffin wax stimulated whole saliva was collected, and samples were stored at -20°C until analysis. Serum and salivary protoporphyrin levels were measured fluorometrically as in a previous study (16).

-Statistical analysis: Kruskal-Wallis H-test with Bonferroni correction was used for comparing salivary and serum PX in controls and patients. Sensitivity and specificity and the corresponding 95% IC were calculated for VELscope and salivary PX as well as combination of VELscope and salivary PX by biopsy examination. Furthermore, mean salivary and serum PX of the groups were correlated by using Pearson s correlation test.

## Results

Patient’s demographic data are shown in  Table 1. The oral cancerous and precancerous lesions showed deep purple to deep brown and dark green colour changes respectively while normal showed pale green. All precancerous and cancerous exhibited some degree of VFL. The protoporphyrin X levels in serum, salivary and tissue were significantly higher in precancerous and cancerous patients as compared to normal healthy individuals (*P*=0.01), furthermore, the levels were also significantly higher in cancerous as compared to precancerous patients ( Table 2, *P*=0.05). Salivary and serum PX levels were highly correlated (R²= 0.72) in all groups. Salivary and tissues PX were moderate correlated (R²=0.68) and high correlation was also found in serum and tissue PX (R²=0.75) in all groups. Sensitivity and specificity values were also calculated to evaluate the validity of VELscope examination and salivary PX in oral precancerous and cancerous lesions. The cutoff value of salivary PX levels 0.39, 0.26, 0.24, 0.11 mg/ml for squamous cell carcinoma, oral leukoplakia, oral lichen planus and normal healthy individuals were taken.

**Table 1:**
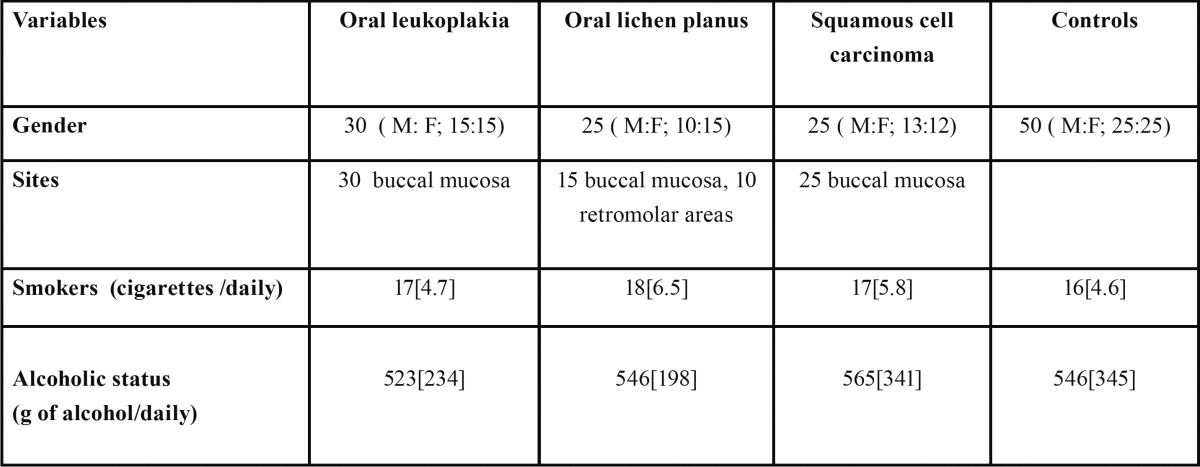
Demographic characteristics of patients and controls.

**Table 2:**
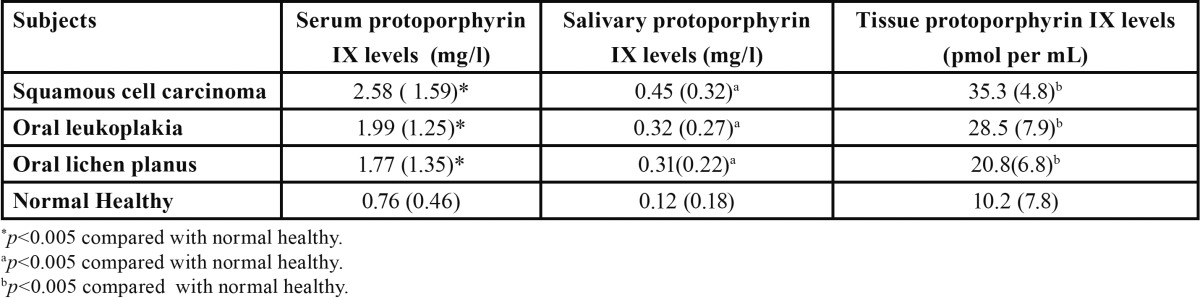
Mean and Standard deviation (SD) Tissue, serum and salivary protoporphyrin IX levels in oral precancerous and cancerous (squamous cell carcinoma, oral leukoplakia, oral lichen planus) and normal healthy subjects.

The sensitivity and specificity to the discrimination of precancerous and cancerous lesions & condition from the healthy tissues were higher by combination approaches of salivary protoporphyrin X and VELscope® system compared to individual approach ( Table 3).

**Table 3:**
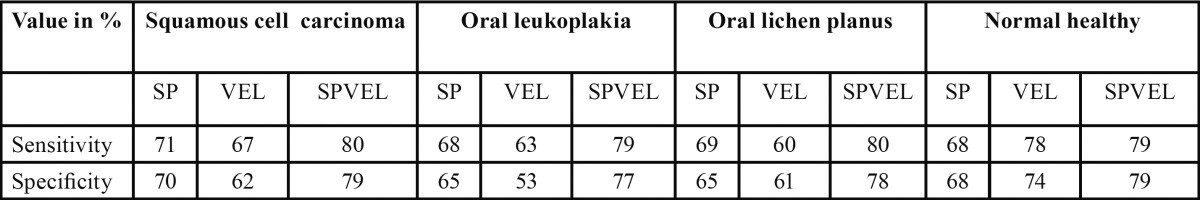
Sensitivity and specificity of salivary protoporphyrin ( SP), VELscope ( VEL), and combination of salivary protoporphyrin and VELscope ( SPVEL) in oral precancerous and cancerous lesions.

## Discussion

The endogenous fluorophores that are most important for optical screening and diagnosis of precancerous and cancerous lesions are those exciting in the spectrum from visible blue (400–450 nm) to UV-A (315–400 nm) with properties spectroscopically correlated to diseased tissues. The concept behind tissue auto-florescence is change in the structure, metabolism of the epithelium, as well as change of the subepithelial stroma which alters their interaction with light (11-18). Endogenous porphyrins have been controversially discussed in literature concerning their tumor-localizing properties (19,20). A most common theory is that, the red fluorescence is a product of microbial porphyrin synthesis and therefore its distribution is limited to the necrotic surface of necrotic tumors (20). The oral cancerous lesions appeared deep purple to deep brown and precancerous appeared dark green while normal showed pale green colour changes. It might be due to higher accumulation of PPIX and a decrease of green auto fluorescence within the precancerous and cancerous lesions. This seems to occur because of a relative lack of ferro-chelatase, an enzyme required to incorporate chelated iron into PP IX to form the heme molecule (11,18). Higher concentration of PIX in serum, tissues and saliva was found in cancerous and pre-cancerous conditions as compared to normal healthy. Tissue, salivary and serum PX levels were highly correlated in all groups. Also, salivary and tissue PX were highly correlated. Therefore, salivary testing, a non-invasive alternative to serum and tissues testing can be considered as an effective modality for diagnosis and for prognosis prediction of various diseases such as oral cancer and pre-cancerous lesions, as well as for monitoring the patient’s post therapy status.

The VELscope is a portable clinical diagnostic tool permitting the direct visualization of the oral cavity and is sold for use in the screening of oral cancer. Oral cancerous and precancerous lesions show deep purple to deep brown and dark green colour changes respectively while normal shows pale green. VELscope achieved a sensitivity of 62.8% and specificity of 62.2% in discriminating clinically evident oral pre-cancerous and cancerous lesions from normal tissue which is lower than previous reports (16,17,18,21-23). The tonality for interpretation purposes is straightforward: pale green for healthy tissues and dark green, brown or black (loss of fluorescence) for a pathological situation. The VELscope is intended to be used by a dentist or health-care provider as an adjunct to traditional oral examination by incandescent light to enhance the visualization of oral mucosal abnormalities that may not be apparent or visible to the naked eye, such as oral cancer or premalignant dysplasia (11,17-23). VELscope is intended to be used by a surgeon to help identify diseased tissue around a clinically apparent lesion and thus aid in determining the appropriate margin for surgical excision. The sensitivity and specificity to the discrimination of precancerous and cancerous lesions from the healthy tissues were higher by combination approaches of salivary protoporphyrin X and VELscope® system as compared to individual approach. Further studies are required to further investigate large samples of oral precancerous and cancerous lesions to test sensitivity and specificity and thus validate the clinical applicability of combination approach of fluorescence imaging and salivary biomarkers in (pre)cancerous diagnostics.
